# Accurate Prediction of Drug Activity by Computational Methods: Importance of Thermal Capacity

**DOI:** 10.3390/molecules30122563

**Published:** 2025-06-12

**Authors:** Luigi Leonardo Palese

**Affiliations:** Department of Translational Biomedicine and Neurosciences—(DiBraiN), University of Bari ‘Aldo Moro’, 70124 Bari, Italy; luigileonardo.palese@uniba.it

**Keywords:** molecular dynamics simulation, protein binding, ligands, thermodynamics, water, heath capacity, HIV, protease

## Abstract

Heat capacity is one of the most important thermodynamic quantities in protein biochemistry. Upon the binding of small molecules, a change in the heat capacity of proteins is generally observed, and this is often used in drug discovery. However, few computational works dedicated to the study of these phenomena are available in the literature. Here, a simple computational method for determining the change in heat capacity upon the binding of small ligands has been evaluated. The method is based on the accurate calibration of the solvent’s thermal properties in the simulation conditions used in order to simply subtract its contribution to calculate the variations in the heat capacity of the system of interest. Using HIV protease as a model system, for which numerous experimental thermodynamic data are available, estimates of the change in heat capacity upon binding were obtained, which were similar to those observed experimentally. Furthermore, the predicted variations in heat capacity appear to be able to discriminate between molecules that behave as effective inhibitors of the enzyme and molecules that are able to bind the enzyme but not inhibit it. The results obtained suggest that this computational approach could be a useful aid in the in silico screening of new ligands for targets of interest.

## 1. Introduction

The study of interactions between proteins and small molecules is one of the most interesting topics in current biochemical research. Although the importance of the computational approach in this field of research has been known for some time [1], the impressive development of hardware, software, and algorithms in recent years has led to a real paradigm shift: the computational approach is no longer just a useful aid to experimental data but has actually become central [2]. Particularly in the field of drug discovery, the computational methods available allow for a significant reduction in research and development times. Using the vast quantities of genomic data, bioinformatics is able to identify genes and, therefore, proteins that are key players in many pathological situations [3,4]. Protein structure prediction methods have reached a level of accuracy that was unthinkable until a few years ago [5,6], greatly expanding the number of potential targets that can be attacked with computational methods [7]. In addition, the sophistication of molecular dynamics simulations [8] allows us to analyze in extreme detail the thermodynamic characteristics of systems of biomedical interest, including the interactions between small molecules and proteins, which are important not only to identify the binding sites between molecules and proteins but also to clarify the mechanism of action of drugs [9]. Moreover, we consider the artificial intelligence algorithms that are now applied in every aspect of the computational study of interactions between proteins and small molecules [10,11].

Alongside the old concept of pharmacophore [12], which was subsequently developed up to modern versions [2,13], and QSAR studies [14], molecular docking [15] is one of the most used computational approaches for the screening of small molecules capable of binding proteins and altering their function. The theoretical foundations of docking are based on the fact that the molecular recognition between target proteins and small molecules is due to both the geometric correspondence and the energetics of the interaction. Typically, a semiflexible approach is used, with the small molecule free to change conformation and a rigid receptor with conformational freedom limited to the rotation of some residues in the binding region (although different approaches are possible, such as rigid docking and fully flexible docking). Molecular dynamics represents a further means to analyze protein–ligand interactions [16]. This is based on classical Newtonian mechanics, which applies empirical force fields to the system of interest, and can be used both to sample the structural landscape of the target protein (important, for example, in molecular docking experiments) and to evaluate the stability [17] and thermodynamics of the protein–ligand interaction [18] or the effects of the ligand itself on the conformational ensemble explored by the protein [19,20].

Heat capacity ($C_{p}$) is one of the most important thermodynamic properties of proteins that can be measured experimentally [21]. Heat capacity changes ($\Delta C_{p}$) can be observed as a consequence of conformational changes, including folding and unfolding, and after binding of small molecules [22,23]. Generally, the sign of heat capacity changes in protein–ligand systems is understood in the sense of changes in polar or apolar interactions. Most theoretical work aimed at explaining heat capacity changes has emphasized the importance of hydration–dehydration phenomena, as well as changes in the conformational equilibrium of proteins. Another possibility that allows us to explain changes in heat capacity after binding is conformational restriction or redistribution of vibrational modes in the protein–ligand complex [24]. From an experimental point of view, calorimetry allows one to evaluate thermodynamic parameters of binding [23], such as Gibbs free energy, enthalpy, and entropy, and thence representing an extremely valuable technique in the research and development of new drug candidates, which is capable of providing useful information, particularly in relation to optimization of the binding enthalpy. Although several factors can sometimes complicate the interpretation of results from calorimetry experiments [25] (also because the thermodynamics of binding is complicated by the fact that no simple and general relationships between ΔG and ΔH or −TΔS are known [26]), this technique is often used to investigate the thermodynamics of small molecule binding to proteins and cooperative phenomena [27,28].

Despite the limitations mentioned above, in many systems of considerable biomedical interest, a clear negative $\Delta C_{p}$ is observed upon the binding of an effective inhibitor to the target protein. As a non-exhaustive example, this type of behavior is well documented experimentally in the case of human thrombin inhibitors [29] and HIV protease inhibitors [30,31]. It would, therefore, be extremely interesting to have reliable computational methods that can predict changes in heat capacity upon the binding of drugs to targets of interest. Interestingly, apart from a seminal work that recently appeared in the literature [32,33], this type of computational technique has been substantially neglected. In this work, a computational method derived from that reported in [33] has been implemented on HIV protease. The data obtained demonstrate the validity of the method in predicting changes in heat capacity following binding, as well as in discriminating effective enzyme ligands from molecules that are able to bind but not inhibit it.

## 2. Results

### 2.1. Theoretical Background

Theoretically, the change in heat capacity following the binding of a ligand to a protein can be obtained by calculating the free energy of the system at different temperatures. This approach could provide a complete set of information on the thermodynamics of the binding due to the fitting with equation [21]

(1) $$\Delta G\left(T\right)=\Delta H\left(T_{ref}\right)-T\Delta S\left(T_{ref}\right)+\Delta C_{p}\left[\left(T-T_{ref}\right)-T\ln\left(T/T_{ref}\right)\right]$$

where $T_{ref}$ is a conveniently chosen reference temperature, would allow us to obtain not only an estimate of the $\Delta C_{p}$ but also of the change in entropy and enthalpy of the system after the binding. Although in principle, this type of approach is valid, in practice, the calculation of free energies through molecular dynamics is affected by errors too large to obtain an accurate binding ΔG, particularly in the case where $\Delta C_{p}$ is different from zero. The change in heat capacity can, however, be obtained without resorting to the calculation of ΔG: at constant pressure, in fact, we have [21,22]

(2) $$C_{p}={\left(\frac{\partial H}{\partial T}\right)}_{p}$$

and therefore it is possible to obtain the change in heat capacity using the simple relation

(3) $$\Delta C_{p}={\left(\frac{\partial H}{\partial T}\right)}_{bound}-{\left(\frac{\partial H}{\partial T}\right)}_{unbound}$$

which requires the calculation of the derivatives with respect to the temperature of the enthalpy in the bound and unbound states [33]. The enthalpy can be replaced by the total average energy or by the total potential energy. A crucial point in this last method is the need to take into account the exact number of degrees of freedom of the system: ideally, the two systems to be compared, bound and unbound, should have the same number of degrees of freedom since each of these contributes to the heat capacity of the system. A possible computational approach is to prepare four systems in a water sphere, namely the protein, the free ligand, the protein–ligand system, and pure water [33]. For each of these systems, the energy is then calculated as a function of temperature. The key point is that all spheres must contain the same number of water molecules, so the number of water molecules that are displaced from the binding site following the interaction with the ligand must be carefully considered.

Setting up a molecular dynamics system such as the one described above requires considerable care in determining the exact number of degrees of freedom of the system. But it is difficult to correctly estimate the number of water molecules that are displaced by the ligand at the binding site: in some cases, it is possible to use crystallographic structures or an estimate must be made as a function of the volume of the ligand or of its degrees of freedom. In any case, different estimates are possible using different methods, and furthermore, changes in the number of water molecules in bulk in the presence or absence of the ligand may not be due only to molecules at the binding site since changes in the protein structure, even subtle, in regions far from the binding site can influence the number of solvent molecules to be considered in this type of system setup.

Taking into account the above considerations, a variation of the algorithm proposed in [33] has been implemented in this work. This is based on the assumption that it is possible to computationally obtain the heat capacity variation after binding using the following equation

(4) $$\Delta C_{p}={\left(\frac{\partial \langle U\rangle }{\partial \langle T\rangle }\right)}_{complex}-\left[{\left(\frac{\partial \langle U\rangle }{\partial \langle T\rangle }\right)}_{protein}+{\left(\frac{\partial \langle U\rangle }{\partial \langle T\rangle }\right)}_{ligand}\right]$$

where 〈U〉 is the average total energy and 〈T〉 is the average temperature of the simulation. The heat capacity of the *i* component (i.e., the protein, the free ligand or the protein–ligand complex) was calculated by subtracting the estimated heat capacity of the aqueous solvent from the total heat capacity of the simulated system *i*:

(5) $${\left(\frac{\partial \langle U\rangle }{\partial \langle T\rangle }\right)}_{i}={\left(\frac{\partial \langle U\rangle }{\partial \langle T\rangle }\right)}_{total}-{\left(\frac{\partial \langle U\rangle }{\partial \langle T\rangle }\right)}_{water}.$$

The heat capacity of water was obtained from a series of separate simulations in which this parameter was calculated using exactly the same simulation setup as the complete system; from these calibration simulations an estimate of the heat capacity to be attributed to each water molecule present under the conditions used was obtained. The interested reader will find the specific simulation conditions in Section 4.

### 2.2. Heat Capacity of Water

**Figure 1:**
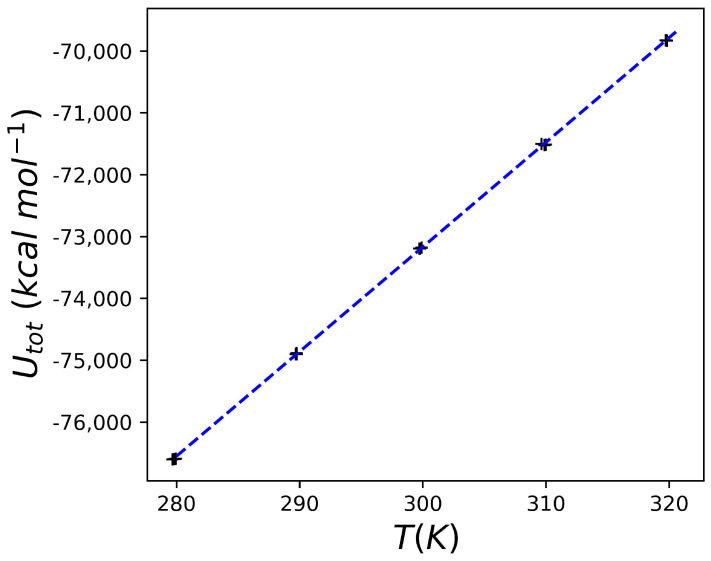
Calculated total energies for pure water. The data reported refer to a series of simulations (three replicates) for a water box containing 9138 water molecules. The line that best fits the data is also shown (the equation has the form $U_{tot}=169.1957\;T-123927.2490$, $R^{2}=0.9999$).

A crucial point for the method used in this work is the accurate calibration of the heat capacity of water. Here, the rigid TIP3P water model was used [34]. To obtain an estimate of the heat capacity, a series of simulations of water boxes with different dimensions (between 60 Å and 66 Å), and therefore a different number of molecules, were carried out. For each of these water boxes, simulations were carried out between 280 K and 320 K (with 10 K intervals), and each of the temperatures was repeated in triplicate. Figure 1 reports one of these series, from which it is possible to appreciate the perfect linearity of the data obtained (the reader should also note the almost perfect superposition of the three replicates at the temperatures explored).

Then, using the derivative Formula (2) from the slope of the best-fitting line and considering the number of water molecules in the simulation, the heat capacity of water is obtained. Using three different boxes (containing 6841, 6845, and 9138 water molecules), we obtain 0.0185 kcal K^−1^ mol^−1^ for all of them. The value is not only in accordance with what has been reported in the literature for computational approaches comparable to the one used in this work (0.0175 and 0.0190 kcal K^−1^ mol^−1^ for spherical and periodic systems respectively [32]) but above all it is perfectly comparable to the experimental value of the heat capacity of water of 0.0180 kcal K^−1^ mol^−1^ [35]. The results obtained for the heat capacity of water allow us not only to calibrate the contribution of the solvent but also the closeness to the experimental data, which indicates that the system here developed, based on a periodic box, Langevin dynamics, and Nose–Hoover thermostat, is certainly reliable in evaluating the heat capacity of a system with a very low deviation from the expected experimental values.

### 2.3. Heat Capacity Calculations of Protein Systems

**Figure 2:**
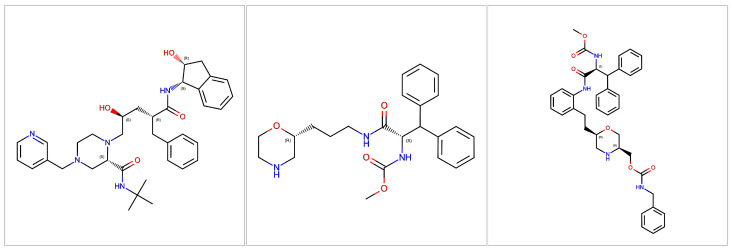
Structures of HIV protease ligands analyzed in this work. On the left is the structure of MK1 (indinavir), in the center 6EH, while the structure on the right is that of 6EF.

Indinavir is one of the most studied inhibitors of the HIV protease. Originally reported as L-735,524 [36], it is listed as MK1 in the PDB, where its interaction with various HIV protease, including mutant forms as well as with the HTLV-1 protease, is reported [37,38,39,40,41,42,43,44,45] (see Figure 2). In high-resolution crystallographic structures, the interactions of this ligand with a series of residues of the protease and some molecules of crystallographic water are clearly visible. Numerous residues of the protease show van der Waals interactions with the ligand. In the 1SDT structure, 96 such interactions are described at a distance of less than 4 Å, as well as a series of hydrogen bonds between MK1 and the protease or crystallographic water molecules, including those of the hydroxyl oxygen O_2_ of MK1 with the four carboxylic oxygens of the ASP25 and ASP25’ residues of the protease.

HIV protease inhibition values reported in BindingDB [46,47] (as $K_{i}$) for this molecule range from 0.07 nM to 27 nM, in line with the efficacy as an inhibitor of the enzyme. Molecular docking (see Section 4) also shows a significant interaction, with a calculated binding energy of −11.89 kcal mol^−1^ using the protein structure reported in 1SDT as a target (see also Table 1 below). The average of all docking experiments performed in this study with MK1 on three different crystallographic structures of the HIV protease (each at least in triplicate) leads us to estimate a $K_{d}$ in the nanomolar range (6.9 nM more precisely), which is perfectly in agreement with the values of the inhibition constant reported in the literature for indinavir.

**Figure 3:**
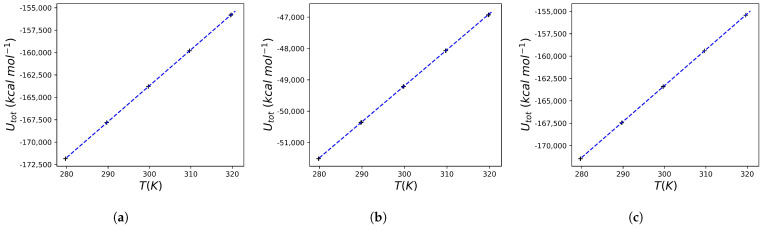
Calculated total energies for the HIV protease and MK1 (indinavir) system. (**a**) The data reported refer to a series of simulations (three replicates) for a water box containing 20,521 water molecules and the HIV protease. The line that best fits the data is also shown (the equation has the form $U_{tot}=401.6620\;T-284184.0648$, $R^{2}=0.9999$). (**b**) The data reported refer to a series of simulations (three replicates) for a water box containing 6172 water molecules and the MK1 molecule. The line that best fits the data is also shown (the equation has the form $U_{tot}=114.9582\;T-83678.3397$, $R^{2}=0.9999$). (**c**) The data reported refer to a series of simulations (three replicates) for a water box containing 20503 water molecules and the HIV protease/MK1 complex. The line that best fits the data is also shown (the equation has the form $U_{tot}=401.4182\;T-283721.8660$, $R^{2}=0.9999$).

In addition to a large amount of crystallographic and inhibition data, experiments on the change in heat capacity as a result of binding are also available for indinavir. In general, as noted above, effective HIV protease inhibitors cause a negative change in the heat capacity upon binding. A $\Delta C_{p}=-0.450\pm 0.050\;$ kcal K^−1^ mol^−1^ is observed for indinavir. Comparable $\Delta C_{p}$ values are reported for nelfinavir, saquinavir and ritonavir (−0.400±0.040, −0.340±0.020, and −0.380±0.030 kcal K^−1^ mol^−1^ respectively) [31]. Using the protocol reported in the Section 4, the heat capacity change for indinavir was calculated: Figure 3 reports the result of the mean total energy as a function of temperature for the HIV protease, for the MK1 molecule in aqueous solution and for the protein bound to MK1 (obtained using the structure reported in 1SDT). The relationships for all conditions are perfectly linear, and from the slopes of each of these graphs, the heat capacity of the system is obtained (see the theoretical justification above). The calculated value is $\Delta C_{p}=-0.5841\;$ kcal K^−1^ mol^−1^, which, although not identical, is nevertheless close to the experimental one, with an error comparable to that reported in other works using a similar approach [33].

Having demonstrated that the computational protocol used in this work gives satisfactory estimates of the heat capacity of both water and the HIV protease/indinavir system, a possible application of this method is to evaluate whether other protease ligands induce $\Delta C_{p}$ comparable to indinavir itself. Many effective HIV protease ligands (so effective that they are used in clinical practice as drugs for the treatment of AIDS), in fact, share a variation in the heat capacity comparable to that observed with indinavir after binding (see above). To evaluate this possibility, two HIV protease ligands [48], very similar from a chemical point of view, whose crystallographic structure and experimental $K_{i}$ values are known, were analyzed (see Figure 2). The first ligand is reported in the PDB as 6EH as part of the structure 5IVQ, whose official chemical name is methyl *N*-[(2*S*)-1-[3-[(2*R*)-morpholin-2-yl]propylamino]-1-oxo-3,3-diphenylpropan-2-yl]carbamate. The second is indicated as 6EF in the PDB and is contained in the structure 5IVS and has the chemical name [(3*S*,6*R*)-6-[2-[2-[[(2*S*)-2-(methoxycarbonylamino)-3,3-diphenylpropanoyl]amino]phenyl]ethyl]morpholin-3-yl]methyl *N*-benzylcarbamate. The latter is a potent in vitro inhibitor of HIV protease, with a reported $IC_{50}$ of 6.1 nM. The ligand 6EH is instead a weak enzyme inhibitor since at 1μM, an inhibition of the activity of 11% is observed. However, this is capable of binding to the active site of the protease, so much so as to obtain a good crystallographic resolution of the interaction, and has represented the starting point for the development of a series of morpholine compounds culminating with MK-8718, which is still under development. Therefore, these two compounds, which belong to the same chemical class of protease inhibitors and are certainly able to bind the enzyme but with a completely different effect in terms of inhibition potency, represent an ideal test set to evaluate the hypothesis that the calculation of the change in heat capacity can be used to provide complementary information to molecular docking on the opportunity to evaluate or not a compound from an experimental point of view.

Molecular docking can be performed in several approaches, but the main distinction is between rigid docking and flexible docking. In flexible docking, the ligand and/or receptor can change their conformation during the computation. This is achieved by identifying rotatable bonds in the ligand and allowing the rigid parts to move during the energy optimization process. In rigid docking, both the ligand and receptor are treated as rigid structures, which means that their conformations remain fixed during the docking simulation. Although much simpler and less computationally demanding than flexible docking, it should be considered only in some special cases where both the ligand and the receptor have rigid characteristics. The choice of the type of docking to be performed, therefore, depends on the research objective and the available information. In this work, a mixed approach was used, in which flexible ligands were challenged with different experimental conformations of the protease modeled as rigid. The use of multiple rigid experimental structures of the receptor avoids introducing artifacts due to the simple rotation of the amino acid side chains in the binding region (docking programs, in general, are not as accurate as molecular dynamics programs), allowing, at the same time, the binding energy to be evaluated both as average and as minimum in the presence of a particular conformation. For validation, redocking of MK1, 6EH, and 6EF was performed on the crystallographic structure of 1SDT, 5IVQ, and 5IVS, respectively. The RMSDs of the best poses, based on the binding energy, are found to be 1.32 Å for MK1 on 1SDT, 5.61 Å for 6EH on 5IVQ (further confirming that it is a poor ligand of HIV protease), and 1.63 Å for 6EF on 5IVS (see Section 4). The results of the molecular docking confirm what is known experimentally and are reported in Table 1. From these data one can also obtain a rough estimate of the dissociation constants: a difference of about two orders of magnitude of the dissociation constants is predicted, placing the $K_{d}$ of 6EF in the nanomolar range, whilst the binding capacity of 6EH is probably overestimated by docking, considering the experimental inhibition values.

**Figure 4:**
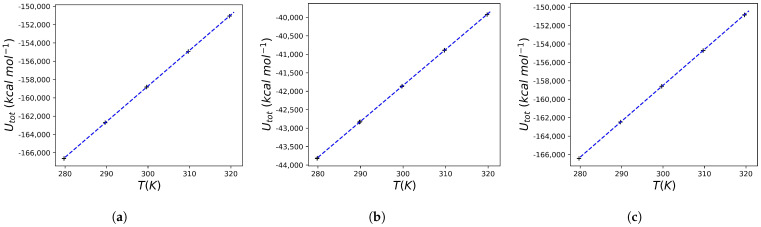
Calculated total energies for the HIV protease and 6EH system. (**a**) The data reported refer to a series of simulations (three replicates) for a water box containing 19,870 water molecules and the HIV protease. The line that best fits the data is also shown (the equation has the form $U_{tot}=389.4320\;T-275541.9406$, $R^{2}=0.9999$). (**b**) The data reported refer to a series of simulations (three replicates) for a water box containing 5236 water molecules and the 6EH molecule. The line that best fits the data is also shown (the equation has the form $U_{tot}=97.4343\;T-71073.8775$, $R^{2}=0.9999$). (**c**) The data reported refer to a series of simulations (three replicates) for a water box containing 19853 water molecules and the HIV protease/6EH complex. The line that best fits the data is also shown (the equation has the form $U_{tot}=389.7589\;T-275415.0402$, $R^{2}=0.9999$).

**Figure 5:**
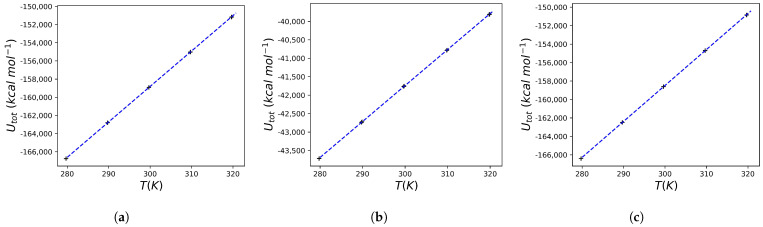
Calculated total energies for the HIV protease and 6EF system. (**a**) The data reported refer to a series of simulations (three replicates) for a water box containing 19,883 water molecules and the HIV protease. The line that best fits the data is also shown (the equation has the form $U_{tot}=389.9753\;T-275808.8660$, $R^{2}=0.9999$). (**b**) The data reported refer to a series of simulations (three replicates) for a water box containing 5226 water molecules and the 6EF molecule. The line that best fits the data is also shown (the equation has the form $U_{tot}=97.4781\;T-70982.6109$, $R^{2}=0.9999$). (**c**) The data reported refer to a series of simulations (three replicates) for a water box containing 19,853 water molecules and the HIV protease/6EF complex. The line that best fits the data is also shown (the equation has the form $U_{tot}=389.5510\;T-275351.4252$, $R^{2}=0.9999$).

The results of the molecular dynamics conducted using the crystallographic structure 5IVQ (and the associated ligand 6EH) and 5IVS (and the associated ligand 6EF) are reported in Figure 4 and Figure 5, respectively. Also, in this case, a perfect linearity of 〈U〉 vs. 〈T〉 is observed, and this allows us to calculate the $C_{p}$ of the various systems (i.e., the protein, the free ligand in solution, and the protein–ligand complex) taking into account the heat capacity of the solvent. The data obtained are extremely interesting: after binding of 6EF to the HIV protease a $\Delta C_{p}=-0.6894\;$ kcal K^−1^ mol^−1^ is predicted, while after binding of 6EH to the enzyme the obtained value is $\Delta C_{p}=+0.1661\;$ kcal K^−1^ mol^−1^. As expected, in the case of the ligand that can not only selectively bind to the protein but also inhibit it at very low concentrations, a *negative* $\Delta C_{p}$ is observed. In contrast, for the ligand that, although it binds to the protein, is a poor inhibitor of the same, a *positive* $\Delta C_{p}$ is observed. This suggests that the proposed computational protocol can provide useful indications on the expected experimental efficacy.

It should also be noted that the structures of the proteins used in this work have some sequence differences. Compared to the sequence of the structures 5IVQ and 5IVS [48], in 1SDT, there are some mutations that are described as an optimization of the wild-type sequence (i.e., they are not mutations involved in resistance phenomena) [41]. The effect of these mutations seems completely negligible for the purposes of this analysis since the partial heat capacity values obtained with the systems containing the protein alone are equal to 21.8104, 21.5111, and 21.6870 kcal K^−1^ mol^−1^ for 5IVS, 5IVQ, and 1SDT respectively (all corresponding to values around 1.0cal K^−1^ g^−1^).

## 3. Discussion

**Figure A1:**
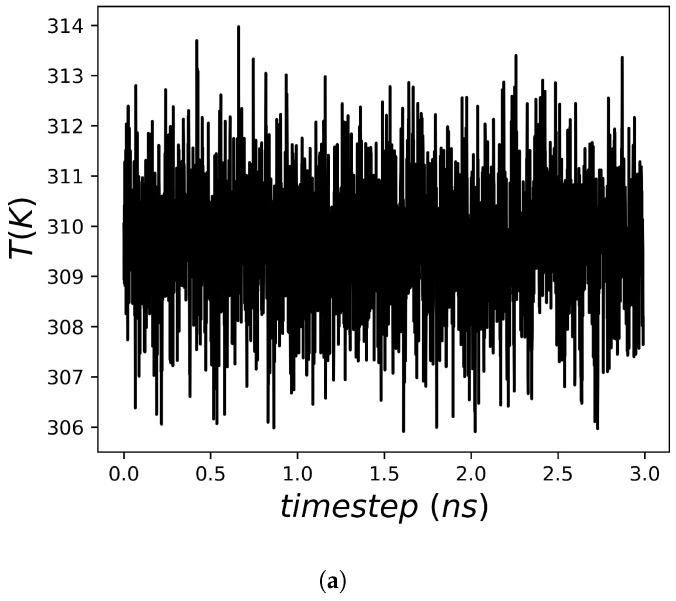

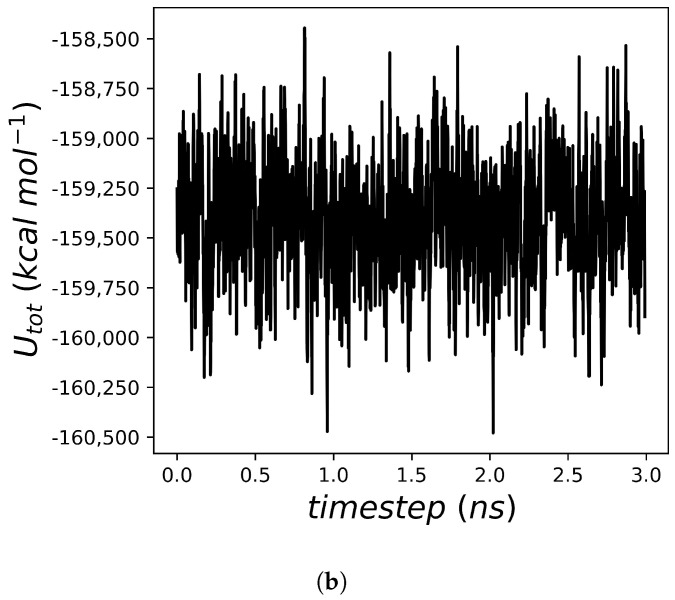
Energy and temperature values obtained during a simulation. The data are those of one of the replicates of the HIV protease—indinavir complex at 310 K. (**a**) Temperature data. (**b**) Total energy data.

**Figure A2:**
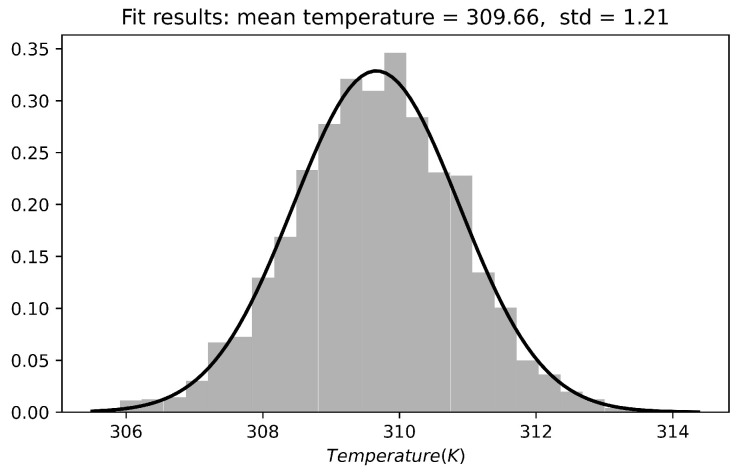
Temperature distribution obtained during a simulation. The data (gray bars) are those of one of the replicates of the HIV protease—indinavir complex at 310 K; the Gaussian fit is reported as black solid line (mean value and standard deviation are reported at the top of the graph).

The aim of this study was to implement a simple computational protocol capable of providing accurate predictions of the heat capacity change following the interaction of a protein of interest with a ligand. The results obtained allow us to state that the approach used leads to state-of-the-art results for the heat capacity of water, which is the (simple) solvent used here. The result is comforting, but it should be emphasized that the computational system, in this case, is exactly pure water. The application of the computational protocol outlined in the Theoretical Background subsection to an HIV protease inhibitor whose binding leads to a known change in the heat capacity of the system led to the prediction of a $\Delta C_{p}$ very similar to the experimental one. It should be emphasized once again that the remarkable aspect is that the value obtained is the result of a computational system that, although accurate in the choice of the chemical-physical simulation parameters, is extremely simplified compared to the experimental or computational calorimetry setup (see for example [31,32]). Complications regarding the exact reproduction of the experimental buffers used in the calorimetry experiments were avoided, with the aim of evaluating the predictive power of a very simple to implement algorithm. The significant simplifications introduced in the algorithm (which avoids the accurate counting of degrees of freedom during the preparation of the computational system) make this approach very convenient to use downstream of in silico screening programs. Moreover, the data obtained suggest that even molecules that give a significant docking score (but that are known to be poor inhibitors of HIV protease, as in the case of the ligand 6EH) lead to a predicted value of $\Delta C_{p}$ that is completely different from what is obtained in the case of good inhibitors of the enzyme (so good as to be interesting also from the point of view of clinical application) such as 6EF. The protocol used here leads to significant results even using relatively short simulation times. This is somewhat expected since the equilibrium or thermal relaxation phenomena are very fast in the time scale of ps [49]. The data obtained here, such as the stability of total energy, the Gaussian fluctuations of the temperature (see Figure A1 and Figure A2 in Appendix A), or the perfect linearity of the observed (Figure 1 and Figure 3, Figure 4 and Figure 5), and theoretically predicted, relation between temperature and total energy, further support this type of computational protocol.

One of the limitations of this study is that the computational protocol does not allow to make certain hypotheses on the exact cause of the variation of $C_{p}$. Experimentally, it has been reported that, from a thermodynamic point of view, the binding strength of the protease with indinavir (MK1) is mainly due to a large positive entropy change with, at most, a small enthalpic contribution [31]. However, these evidences of “in bulk” thermodynamics leave open several possible microscopic explanations for the variation of thermal capacity observed experimentally and computationally. One possibility is that $\Delta C_{p}$ is due to variations in protein mobility, in the sense that a greater or lesser rigidity of the system could explain the variations in heat capacity. The data of the root mean squared fluctuations (RMSF) in the various conditions would seem to suggest that this cannot be a plausible explanation since no statistically significant differences are observed between the various conditions (free protein and bound protein). Another possibility is that changes in heat capacity can be explained in terms of conformational equilibria [23,32,50]. In future studies, this type of approach will be used to analyze the microscopic foundations of the variations in heat capacity of proteins following binding.

## 4. Materials and Methods

The structural models for HIV protease were based on crystal structures reported in the PDB [51,52] entries 1SDT, 5IVQ, and 5IVS, which also contain the ligands denoted MK1, 6EH, and 6EF, respectively [41,48]. Structure parameterization was carried out using CHARMM-GUI [53,54,55]. Ligand parameterization was performed as described in [56] using the CHARMM General Force Field to generate the topology and parameter files starting from the SDF file in the PDB. The simulation systems included the three protease-ligand complexes, the proteases, and the three ligands free in water. The structure of apo-protease was obtained from the 1SDT, 5IVQ, and 5IVS structures by removing the ligand from the binding site and all other components in the crystal structures. Similarly, free ligands were obtained by retaining only the molecules prior to water-box build-up. In the reported simulations, histidines were modeled with the δ nitrogen protonated, while the aspartic acid of the catalytic center (ASP25) was modeled as protonated in the A subunit and deprotonated in the B subunit. All of these systems were immersed in a water box with at least 15 Å of padding. Plain water cubic systems with side dimensions equal to 60 Å or 66 Å have been obtained using the same setup.

Molecular dynamics has been performed in NAMD (version 2.14) [57,58] using the CHARMM36m force field [59], periodic boundary conditions and particle-mesh Ewald (PME). The TIP3P water model [34] was used; 1–3 pairs of bonded atoms were excluded from non-bonded interactions, and van der Waals interactions were truncated at 12 Å using the built-in NAMD function that truncates the van der Waals potential energy smoothly at the cutoff distance (*switchdist* parameter 10.0). Electrostatics and van der Waals interactions were calculated for all pairs of atoms whose distance was less than or equal to 14 Å, and the reassignment of the atom lists for the calculation of non-covalent interactions was performed every 10 time steps. Short-range non-bonded interactions were evaluated at each time step, while long-range electrostatic interaction forces were evaluated every two time steps. After 1000 conjugate gradient minimization steps, systems were equilibrated in the NpT ensemble for 1 ns with a 2 fs time step at a fixed temperature using Langevin dynamics; a Langevin coupling coefficient equal to 1/ps was applied to all atoms, but excluding hydrogens. Langevin piston Nose–Hoover method in NAMD was used for pressure control [60,61] (target pressure 1.01325 bar, barostat oscillation time scale 100 fs, barostat damping time scale 50 fs). After equilibration, the system was simulated for 3 ns under the same NpT conditions, but using a 1 fs time step and with the hydrogen–oxygen and hydrogen–hydrogen distances in waters constrained to the nominal length or angle given in the TIP3P model. Production runs were performed in triplicate for each temperature.

Structural analysis was performed in VMD (version 1.9.3) [62]. The average total energy 〈U〉 and the average temperature 〈T〉 for each simulation were obtained from the NAMD log file of the production run (sampling frequency 1 ps^−1^). An example of the data used for heat capacity calculations is given in Appendix A. Figure A1 reports the temperature and total energy values obtained from the output of the molecular dynamics program (conventionally referred to as the log file in NAMD). Figure A2 shows that temperature fluctuations during simulation can be fitted with a Gaussian. The simulation reported is one of three obtained using the system consisting of HIV protease (obtained from the 1SDT structure) bound to MK1 at 310 K. The corresponding log file is reported as a zip file in the Supplementary Materials (file 1SDT-MK1-310-run1.log). The interested reader can use this log file in the VMD plugin NAMD plot and extract the total energy and temperature data (as well as all other data recorded in the log file) to reproduce the Figure A1 and Figure A2 in Appendix A.

**Figure A3:**
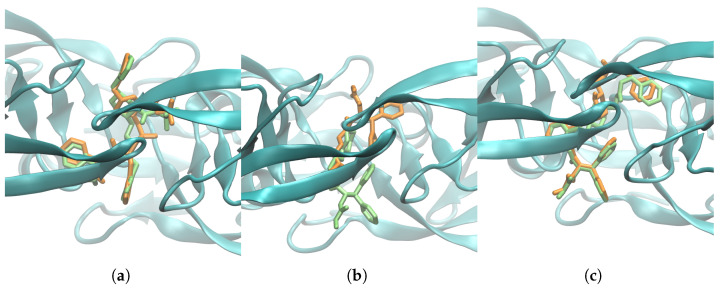
Redocking of the ligands. The crystallographic ligand is represented in lime, while the pose obtained by molecular docking is reported in orange. (**a**) MK1 and 1SDT. (**b**) 6EH and 5IVQ. (**c**) 6EF and 5IVS.

Molecular docking was performed using AutoDock Vina (version 1.2.3) [63,64]. Protein structure files in *pdbqt* format were obtained by the AutoDockTools suite [65] from the PDB files after the addition of hydrogen atoms and Gasteiger-Marsili charges [66]. Ligand *pdbqt* files for docking were generated by means of the AutoDockTools (version 1.5.6) software using the SDF files stored in the PDB as starting template [65]. For validation by redocking (see Figure A3), the distance between the crystallographic ligand and the docked pose was calculated by DockRMSD [67] after converting the relevant files into *mol2* format via Open Babel (version 3.0.1) [68]. Numerical calculations were performed in Numpy [69]. Graphs were obtained in Matplotlib (version 3.5.2) [70].

## Figures and Tables

**Table 1:** Molecular docking results. Reported values are the calculated binding energies ^1^ of various ligands using different HIV protease structures as target.

Ligand ID	1SDT	5IVQ	5IVS
MK1	−11.89 (0.20)	−10.16 (0.21)	−11.33 (0.26)
6EF	−10.93 (0.40)	−10.79 (0.47)	−11.73 (0.04)
6EH	−9.35 (0.03)	−9.15 (0.15)	−9.41 (0.47)

^1^ Reported values are averages of three independent determinations ± SD (kcal mol^−1^).

## Data Availability

All data relevant to this publication are reported in the work. Further data may be provided by the author upon reasonable request.

## Supplementary Materials

The following supporting information can be downloaded at: https://www.mdpi.com/article/10.3390/molecules30122563/s1. The file contains the raw data needed to reproduce the Figure A1 and Figure A2 in Appendix A.

## Abbreviations

The following abbreviations are used in this manuscript:

AIDS	Acquired ImmunoDeficiency Syndrome
PDB	Protein Data Bank
PME	Particle-Mesh Ewald
RMSD	Root Mean Squared Deviations
RMSF	Root Mean Squared Fluctuations
SDF	Structured Data Files

## References

[1] Jorgensen W.L. (2004). The many roles of computation in drug discovery. Science.

[2] Lin X., Li X., Lin X. (2020). A review on applications of computational methods in drug screening and design. Molecules.

[3] Yamanishi Y., Araki M., Gutteridge A., Honda W., Kanehisa M. (2008). Prediction of drug–target interaction networks from the integration of chemical and genomic spaces. Bioinformatics.

[4] Bakheet T.M., Doig A.J. (2009). Properties and identification of human protein drug targets. Bioinformatics.

[5] Jumper J., Evans R., Pritzel A., Green T., Figurnov M., Ronneberger O., Tunyasuvunakool K., Bates R., Žídek A., Potapenko A. (2021). Highly accurate protein structure prediction with AlphaFold. Nature.

[6] Abramson J., Adler J., Dunger J., Evans R., Green T., Pritzel A., Ronneberger O., Willmore L., Ballard A.J., Bambrick J. (2024). Accurate structure prediction of biomolecular interactions with AlphaFold 3. Nature.

[7] Borkakoti N., Thornton J.M. (2023). AlphaFold2 protein structure prediction: Implications for drug discovery. Curr. Opin. Struct. Biol..

[8] Karplus M. (2014). Development of Multiscale Models for Complex Chemical Systems: From H+ H 2 to Biomolecules (Nobel Lecture). Angew. Chem. Int. Ed. Engl..

[9] De Vivo M., Masetti M., Bottegoni G., Cavalli A. (2016). Role of molecular dynamics and related methods in drug discovery. J. Med. Chem..

[10] Qi X., Zhao Y., Qi Z., Hou S., Chen J. (2024). Machine learning empowering drug discovery: Applications, opportunities and challenges. Molecules.

[11] Deng J., Yang Z., Ojima I., Samaras D., Wang F. (2022). Artificial intelligence in drug discovery: Applications and techniques. Brief. Bioinform..

[12] Ehrlich P. (1909). Über den jetzigen Stand der Chemotherapie. Ber. Dtsch. Chem. Ges..

[13] Yang S.Y. (2010). Pharmacophore modeling and applications in drug discovery: Challenges and recent advances. Drug Discov. Today.

[14] Muratov E.N., Bajorath J., Sheridan R.P., Tetko I.V., Filimonov D., Poroikov V., Oprea T.I., Baskin I.I., Varnek A., Roitberg A. (2020). QSAR without borders. Chem. Soc. Rev..

[15] Paggi J.M., Pandit A., Dror R.O. (2024). The art and science of molecular docking. Annu. Rev. Biochem..

[16] Guterres H., Im W. (2020). Improving protein-ligand docking results with high-throughput molecular dynamics simulations. J. Chem. Inf. Model..

[17] Sardanelli A.M., Isgrò C., Palese L.L. (2021). SARS-CoV-2 main protease active site ligands in the human metabolome. Molecules.

[18] Limongelli V. (2020). Ligand binding free energy and kinetics calculation in 2020. Wiley Interdiscip. Rev. Comput. Mol. Sci..

[19] Miller D.W., Dill K.A. (1997). Ligand binding to proteins: The binding landscape model. Protein Sci..

[20] Ikeguchi M., Ueno J., Sato M., Kidera A. (2005). Protein structural change upon ligand binding: Linear response theory. Phys. Rev. Lett..

[21] Prabhu N.V., Sharp K.A. (2005). Heat capacity in proteins. Annu. Rev. Phys. Chem..

[22] Cooper A. (2010). Protein heat capacity: An anomaly that maybe never was. J. Phys. Chem. Lett..

[23] Vega S., Abian O., Velazquez-Campoy A. (2016). On the link between conformational changes, ligand binding and heat capacity. Biochim. Biophys. Acta-Gen. Subj..

[24] Cooper A., Johnson C.M., Lakey J.H., Nöllmann M. (2001). Heat does not come in different colours: Entropy–enthalpy compensation, free energy windows, quantum confinement, pressure perturbation calorimetry, solvation and the multiple causes of heat capacity effects in biomolecular interactions. Biophys. Chem..

[25] Geschwindner S., Ulander J., Johansson P. (2015). Ligand binding thermodynamics in drug discovery: Still a hot tip?. J. Med. Chem..

[26] Reynolds C.H., Holloway M.K. (2011). Thermodynamics of ligand binding and efficiency. ACS Med. Chem. Lett..

[27] Weber P.C., Salemme F.R. (2003). Applications of calorimetric methods to drug discovery and the study of protein interactions. Curr. Opin. Struct. Biol..

[28] Abian O., Vega S., Velazquez-Campoy A. (2023). Biological calorimetry: Old friend, new insights. Biophysica.

[29] Winquist J., Geschwindner S., Xue Y., Gustavsson L., Musil D., Deinum J., Danielson U.H. (2013). Identification of structural–kinetic and structural–thermodynamic relationships for thrombin inhibitors. Biochemistry.

[30] Todd M.J., Freire E. (1999). The effect of inhibitor binding on the structural stability and cooperativity of the HIV-1 protease. Proteins.

[31] Todd M.J., Luque I., Velázquez-Campoy A., Freire E. (2000). Thermodynamic basis of resistance to HIV-1 protease inhibition: Calorimetric analysis of the V82F/I84V active site resistant mutant. Biochemistry.

[32] Åqvist J., van der Ent F. (2022). Calculation of heat capacity changes in enzyme catalysis and ligand binding. J. Chem. Theory Comput..

[33] Koenekoop L., Åqvist J. (2024). Computational Analysis of Heat Capacity Effects in Protein–Ligand Binding. J. Chem. Theory Comput..

[34] Jorgensen W.L., Chandrasekhar J., Madura J.D., Impey R.W., Klein M.L. (1983). Comparison of simple potential functions for simulating liquid water. J. Chem. Phys..

[35] Weast R.C. (1986). CRC Handbook of Chemistry and Physics.

[36] Vacca J.P., Dorsey B., Schleif W., Levin R., McDaniel S., Darke P., Zugay J., Quintero J., Blahy O., Roth E. (1994). L-735,524: An orally bioavailable human immunodeficiency virus type 1 protease inhibitor. Proc. Natl. Acad. Sci. USA.

[37] Chen Z., Li Y., Chen E., Hall D.L., Darke P.L., Culberson C., Shafer J.A., Kuo L.C. (1994). Crystal structure at 1.9-A resolution of human immunodeficiency virus (HIV) II protease complexed with L-735,524, an orally bioavailable inhibitor of the HIV proteases. J. Biol. Chem..

[38] Munshi S., Chen Z., Li Y., Olsen D.B., Fraley M.E., Hungate R.W., Kuo L.C. (1998). Rapid X-ray diffraction analysis of HIV-1 protease–inhibitor complexes: Inhibitor exchange in single crystals of the bound enzyme. Acta Crystallogr. D.

[39] Munshi S., Chen Z., Yan Y., Li Y., Olsen D.B., Schock H.B., Galvin B.B., Dorsey B., Kuo L.C. (2000). An alternate binding site for the P1–P3 group of a class of potent HIV-1 protease inhibitors as a result of concerted structural change in the 80s loop of the protease. Acta Crystallogr. D.

[40] King N.M., Melnick L., Prabu-Jeyabalan M., Nalivaika E.A., Yang S.S., Gao Y., Nie X., Zepp C., Heefner D.L., Schiffer C.A. (2002). Lack of synergy for inhibitors targeting a multi-drug-resistant HIV-1 protease. Protein Sci..

[41] Mahalingam B., Wang Y.F., Boross P.I., Tozser J., Louis J.M., Harrison R.W., Weber I.T. (2004). Crystal structures of HIV protease V82A and L90M mutants reveal changes in the indinavir-binding site. Eur. J. Biochem..

[42] Clemente J.C., Moose R.E., Hemrajani R., Whitford L.R., Govindasamy L., Reutzel R., McKenna R., Agbandje-McKenna M., Goodenow M.M., Dunn B.M. (2004). Comparing the accumulation of active-and nonactive-site mutations in the HIV-1 protease. Biochemistry.

[43] Liu F., Boross P.I., Wang Y.F., Tozser J., Louis J.M., Harrison R.W., Weber I.T. (2005). Kinetic, stability, and structural changes in high-resolution crystal structures of HIV-1 protease with drug-resistant mutations L24I, I50V, and G73S. J. Mol. Biol..

[44] Coman R.M., Robbins A.H., Fernandez M.A., Gilliland C.T., Sochet A.A., Goodenow M.M., McKenna R., Dunn B.M. (2008). The contribution of naturally occurring polymorphisms in altering the biochemical and structural characteristics of HIV-1 subtype C protease. Biochemistry.

[45] Kuhnert M., Steuber H., Diederich W.E. (2014). Structural basis for HTLV-1 protease inhibition by the HIV-1 protease inhibitor indinavir. J. Med. Chem..

[46] Chen X., Liu M., Gilson M.K. (2001). BindingDB: A web-accessible molecular recognition database. Comb. Chem. High Throughput Screen..

[47] Liu T., Hwang L., Burley S.K., Nitsche C.I., Southan C., Walters W.P., Gilson M.K. (2025). BindingDB in 2024: A FAIR knowledgebase of protein-small molecule binding data. Nucleic Acids Res..

[48] Bungard C.J., Williams P.D., Ballard J.E., Bennett D.J., Beaulieu C., Bahnck-Teets C., Carroll S.S., Chang R.K., Dubost D.C., Fay J.F. (2016). Discovery of MK-8718, an HIV protease inhibitor containing a novel morpholine aspartate binding group. ACS Med. Chem. Lett..

[49] Lervik A., Bresme F., Kjelstrup S., Bedeaux D., Rubi J.M. (2010). Heat transfer in protein–water interfaces. Phys. Chem. Chem. Phys..

[50] Eftink M.R., Anusiem A., Biltonen R.L. (1983). Enthalpy-entropy compensation and heat capacity changes for protein-ligand interactions: General thermodynamic models and data for the binding of nucleotides to ribonuclease A. Biochemistry.

[51] Berman H.M., Westbrook J., Feng Z., Gilliland G., Bhat T.N., Weissig H., Shindyalov I.N., Bourne P.E. (2000). The Protein Data Bank. Nucleic Acids Res..

[52] Burley S.K., Bhikadiya C., Bi C., Bittrich S., Chen L., Crichlow G.V., Christie C.H., Dalenberg K., Di Costanzo L., Duarte J.M. (2021). RCSB Protein Data Bank: Powerful new tools for exploring 3D structures of biological macromolecules for basic and applied research and education in fundamental biology, biomedicine, biotechnology, bioengineering and energy sciences. Nucleic Acids Res..

[53] Jo S., Kim T., Iyer V.G., Im W. (2008). CHARMM-GUI: A web-based graphical user interface for CHARMM. J. Comput. Chem..

[54] Brooks B.R., Brooks C.L., Mackerell A.D., Nilsson L., Petrella R.J., Roux B., Won Y., Archontis G., Bartels C., Boresch S. (2009). CHARMM: The biomolecular simulation program. J. Comput. Chem..

[55] Lee J., Cheng X., Swails J.M., Yeom M.S., Eastman P.K., Lemkul J.A., Wei S., Buckner J., Jeong J.C., Qi Y. (2016). CHARMM-GUI Input Generator for NAMD, GROMACS, AMBER, OpenMM, and CHARMM/OpenMM Simulations Using the CHARMM36 Additive Force Field. J. Chem. Theory Comput..

[56] Kim S., Lee J., Jo S., Brooks C.L., Lee H.S., Im W. (2017). CHARMM-GUI ligand reader and modeler for CHARMM force field generation of small molecules. J. Comput. Chem..

[57] Phillips J.C., Hardy D.J., Maia J.D., Stone J.E., Ribeiro J.V., Bernardi R.C., Buch R., Fiorin G., Hénin J., Jiang W. (2020). Scalable molecular dynamics on CPU and GPU architectures with NAMD. J. Chem. Phys..

[58] Kalé L., Skeel R., Bhandarkar M., Brunner R., Gursoy A., Krawetz N., Phillips J., Shinozaki A., Varadarajan K., Schulten K. (1999). NAMD2: Greater scalability for parallel molecular dynamics. J. Comput. Phys..

[59] Huang J., Rauscher S., Nawrocki G., Ran T., Feig M., De Groot B.L., Grubmüller H., MacKerell A.D. (2017). CHARMM36m: An improved force field for folded and intrinsically disordered proteins. Nat. Methods.

[60] Martyna G.J., Tobias D.J., Klein M.L. (1994). Constant pressure molecular dynamics algorithms. J. Chem. Phys.

[61] Feller S.E., Zhang Y., Pastor R.W., Brooks B.R. (1995). Constant pressure molecular dynamics simulation: The Langevin piston method. J. Chem. Phys.

[62] Humphrey W., Dalke A., Schulten K. (1996). VMD: Visual molecular dynamics. J. Mol. Graph..

[63] Trott O., Olson A.J. (2010). AutoDock Vina: Improving the speed and accuracy of docking with a new scoring function, efficient optimization, and multithreading. J. Comput. Chem..

[64] Eberhardt J., Santos-Martins D., Tillack A.F., Forli S. (2021). AutoDock Vina 1.2.0: New docking methods, expanded force field, and python bindings. J. Chem. Inf. Model..

[65] Morris G.M., Huey R., Lindstrom W., Sanner M.F., Belew R.K., Goodsell D.S., Olson A.J. (2009). AutoDock4 and AutoDockTools4: Automated docking with selective receptor flexibility. J. Comput. Chem..

[66] Gasteiger J., Marsili M. (1978). A new model for calculating atomic charges in molecules. Tetrahedron Lett..

[67] Bell E.W., Zhang Y. (2019). DockRMSD: An open-source tool for atom mapping and RMSD calculation of symmetric molecules through graph isomorphism. J. Cheminform..

[68] O’Boyle N.M., Banck M., James C.A., Morley C., Vandermeersch T., Hutchison G.R. (2011). Open Babel: An open chemical toolbox. J. Cheminform..

[69] Harris C.R., Millman K.J., Van Der Walt S.J., Gommers R., Virtanen P., Cournapeau D., Wieser E., Taylor J., Berg S., Smith N.J. (2020). Array programming with NumPy. Nature.

[70] Hunter J.D. (2007). Matplotlib: A 2D graphics environment. Comput. Sci. Eng..

